# Brain Tumor Detection and Classification Using Deep Learning and Sine-Cosine Fitness Grey Wolf Optimization

**DOI:** 10.3390/bioengineering10010018

**Published:** 2022-12-22

**Authors:** Hanaa ZainEldin, Samah A. Gamel, El-Sayed M. El-Kenawy, Amal H. Alharbi, Doaa Sami Khafaga, Abdelhameed Ibrahim, Fatma M. Talaat

**Affiliations:** 1 Computer Engineering and Control Systems Department, Faculty of Engineering, Mansoura University, Mansoura 35516, Egypt; 2 Department of Communications and Electronics, Delta Higher Institute of Engineering and Technology, Mansoura 35111, Egypt; 3 Department of Computer Sciences, College of Computer and Information Sciences, Princess Nourah bint Abdulrahman University, P.O. Box 84428, Riyadh 11671, Saudi Arabia; 4 Machine Learning & Information Retrieval Department, Faculty of Artificial Intelligence, Kafrelsheikh University, Kafrelsheikh 33511, Egypt

**Keywords:** brain tumor, diagnosing, convolutional neural network, optimization, hyperparameters, deep learning technique

## Abstract

Diagnosing a brain tumor takes a long time and relies heavily on the radiologist’s abilities and experience. The amount of data that must be handled has increased dramatically as the number of patients has increased, making old procedures both costly and ineffective. Many researchers investigated a variety of algorithms for detecting and classifying brain tumors that were both accurate and fast. Deep Learning (DL) approaches have recently been popular in developing automated systems capable of accurately diagnosing or segmenting brain tumors in less time. DL enables a pre-trained Convolutional Neural Network (CNN) model for medical images, specifically for classifying brain cancers. The proposed Brain Tumor Classification Model based on CNN (BCM-CNN) is a CNN hyperparameters optimization using an adaptive dynamic sine-cosine fitness grey wolf optimizer (ADSCFGWO) algorithm. There is an optimization of hyperparameters followed by a training model built with Inception-ResnetV2. The model employs commonly used pre-trained models (Inception-ResnetV2) to improve brain tumor diagnosis, and its output is a binary 0 or 1 (0: Normal, 1: Tumor). There are primarily two types of hyperparameters: (i) hyperparameters that determine the underlying network structure; (ii) a hyperparameter that is responsible for training the network. The ADSCFGWO algorithm draws from both the sine cosine and grey wolf algorithms in an adaptable framework that uses both algorithms’ strengths. The experimental results show that the BCM-CNN as a classifier achieved the best results due to the enhancement of the CNN’s performance by the CNN optimization’s hyperparameters. The BCM-CNN has achieved 99.98% accuracy with the BRaTS 2021 Task 1 dataset.

## 1. Introduction

Recently, digital medical images have been essential for detecting numerous illnesses. It is additionally used for training and research. The need for electronic medical images is growing dramatically; for example, in 2002, the Department of Radiology at the University Hospital of Geneva produced between 12,000 and 15,000 images daily [1]. An efficient and exact computer-aided diagnostic system is required for medical report creation and medical image research. The old method of manually evaluating medical imaging is time-consuming, inaccurate, and prone to human error. Over the medical diseases, the brain tumor has become a serious issue, ranking 10th among the major causes of death in the US. It is reported that 700,000 persons have brain tumors, of which 80 percent are benign and 20 percent are malignant [2]. According to estimates by the American Cancer Society from 2021, 78,980 adults have been diagnosed with a brain tumor, with 55,150 noncancerous and 24,530 malignant tumors (13,840 men and 10,690 females) [3]. According to studies, brain tumor is the top cause of cancer deaths in children and adults worldwide [4].

The most typical kind of brain disease is a brain tumor. It is an unregulated development of brain cells. Brain tumors are always classified into brain tumors, both primary and secondary. The first starts in the brain and usually stays there, whereas the latter starts as cancer somewhere else in the body and spreads to the brain [5]. There are two different forms of tumors: malignant and benign. A benign tumor is a slow-growing tumor that does not infiltrate nearby tissues; on the other hand, a malignant which is a very aggressive tumor that spreads from one location to another. The World Health Organization (WHO) grades a brain tumor as I-IV. Tumors in categories I and II are regarded as slow-growing, while tumors in categories III and IV are always malignant and have a worse prognosis [6].

In recent decades, many imaging techniques such as X-ray, Magneto Encephalo Graphy (MEG), Computed Tomography (CT), Ultrasonography, Electro Encephalo Graphy (EEG), Single-Photon Emission Computed Tomography (SPECT), Positron Emission Tomography (PET), and Magnetic Resonance Imaging (MRI) have emerged that not only exhibit the detailed and complete facets of brain tumors but also help doctors to accurately diagnose the tumor and determine the correct treatment mechanism [4]. MRI is considered the most popular imaging technique for detecting brain tumors [7]. Without subjecting the patients to excessive ionization radiation, MRI is a non and excellent soft tissue contrast imaging technique that gives essential information about brain tumor shape, location, and size.

The brain tumor diagnosis is highly time intensive and largely depends on the radiologist’s skills and knowledge. Because there are more patients, the amount of data that must be processed has grown significantly, making traditional techniques cost and incorrect [8]. The difficulties are associated with significant brain tumor size, shape, and intensity variations for the same tumor type and similar manifestations of other disease types. A misclassification of a brain tumor can result in major consequences and reduce the patient’s survivability. There is a rise in interest in building automated technologies for processing images to overcome the limitations of manual diagnosis [4,9] and other related applications [10,11,12]. Several systems for computer-aided diagnosis (CAD) have been created recently to diagnose brain tumors automatically.

In recent years, among many other applications, artificial intelligence (AI) has demonstrated promising results as a decision support system to assist in the detection of diseases and the establishment of precise medical diagnoses. In order to address practical problems researchers and governments focus on machine learning, a branch of artificial intelligence [13,14]. Machine learning, for instance, may predict the COVID-19 outbreak in the COVID-19 pandemic challenge by determining how risky the virus is and then scaling up the level of the methods performed. In the realm of medical analysis, machine learning algorithms are frequently used for things such as COVID-19 prediction [15], Alzheimer’s disease progression [16], brain tumor development [17], breast cancer progression [18], and other disorders [19,20,21]. Deep learning and machine learning are essential for identifying diseases and resolving medical problems.

Many researchers investigated numerous algorithms for detecting and classifying brain tumors with high performance and less error. Deep Learning (DL) techniques have recently been widely employed to build automatic systems that can accurately classify or segment brain tumors in less time. DL enables the use of a pre-trained Convolutional Neural Network (CNN) model [22] for medical imagery, specifically for the classification of brain tumors, which has been created for various applications, including GoogLeNet [23], AlexNet, and ResNet-34 [24]. DL is made up of a multi-layered deep neural network [25]. The backpropagation algorithm is used by a neural network (NN) to reduce the error between the target and actual value. Nevertheless, even as the number of layers increases, developing artificial neural network models gets more difficult.

The main contributions of the current work are:

- Introduce an enhanced model to improve brain tumor diagnosis.
- It proposes a Brain Tumor Classification Model (BCM-CNN) based on an advanced 3D model using Enhanced Convolutional Neural Network (BCM-CNN).
- The proposed Brain Tumor Classification Model (BCM-CNN) is based on two submodules; (i) CNN hyperparameters optimization using an adaptive dynamic sine-cosine fitness grey wolf optimizer (ADSCFGWO) algorithm followed by trained Model, and (ii) segmentation model.
- The ADSCFGWO algorithm draws from both the sine cosine and grey wolf algorithms in an adaptable framework that uses both algorithms’ strengths.
- The experimental results show that the BCM-CNN as a classifier achieved the best results due to the enhancement of the CNN’s performance by the CNN optimization’s hyperparameters.

The remainder of the paper is structured as follows: A brief review of state-of-the-art deep learning methods for finding brain tumors is discussed in Section 2. The proposed technique is described in detail in Section 3. Section 4 depicts simulation and experimental results. The conclusion and future works are presented in Section 5.

## 2. Related Work

This section introduces a collection of cutting-edge DL-based brain tumor classification techniques. Based on DL and transfer learning algorithms, there are numerous methods for classifying brain tumors. State-of-the-art techniques can be classified into deep learning-based, machine learning-based, and hybrid-based techniques. Table 1 summarizes different classification techniques for a brain tumor.

### 2.1. Deep Learning-Based Techniques

B. Srikanth et al. presented [26] a 16-layer VGG-16 deep NN, which accepts improved images from a prior pre-processing phase as input and moves them through the convolution layer for extracting the features and downsampling (Convolution, ReLU, Max-Pooling). Their proposed approach increased the precision of brain tumor MR image multi-classification. To avoid the overfitting problem, completely linked and SoftMax layers are employed. Lastly, after 20 training iterations, their proposed model achieves the best outcomes, which have a 98 percent accuracy.

GS Tandel et al. [27] The researcher developed five clinical multiclass datasets. They used a transfer learning-based Convolutional Neural Network (CCN) to improve performance in brain tumor classification by employing MRI images. The proposed CNN model was compared to six alternative ML classification approaches, including Decision Tree (DT), Naive Bayes (NB), Linear Discrimination (LD), K-nearest Neighbor, and Support Vector Machine (SVM). The five types of multiclass classification brain tumor datasets are considered, and the proposed CNN-based (DL) model technique beats the six types of machine learning model techniques. For the five classes, the CNN-based AlexNet achieved a mean accuracy rate of 87.14, 93.74, 95.97, 96.65, and 100 percent using three different cross-validation procedures, K2, K5, and K10, respectively.

The authors in [28] proposed a CNN technique for a three-class classification to distinguish between three kinds of brain tumors, including glioma, meningioma, and pituitary tumors. They used a pre-trained GoogleNet for feature extraction from brain MRI scans. To identify the extracted features, proven-based classifications are used. The suggested approach outperforms existing approaches with an average classification accuracy of 98%. Precision, F-score, recall, specificity, and the Area Under the Curve (AUC) are performance metrics employed in the study. According to the result of the research, transfer learning techniques is a highly effective strategy when medical pictures are scarce.

Regarding a three-class brain tumor classification, ref. [29] suggested a deep inception residual network. They have adjusted the output layer of the ResNet V2 network with a dense network and a softmax layer. The suggested model maximizes brain tumor classification accuracy. The proposed model was tested on a publicly accessible brain tumor imaging dataset with 3064 pictures. The accuracy of the proposed model exceeds state-of-the-art techniques by 99.69%.

Using the concept of transfer learning, ref. [30] presented a brain tumor classification technique with MRI images. VGG16, ResNet50, DenseNet, and VGG19 networks use transfer learning to detect the most frequent brain cancers. Deep transfer learning algorithms are trained and evaluated on the publicly available Figshare dataset, which contains 3064 T1-weighted MRI scans from 233 patients with three common brain tumor types: glioma (1426 pictures), pituitary tumor (930 images), and meningioma (708 photos). The suggested model enhances the classification performance by 99.02% compared to ResNet50 and Adadelta.

The RCNN approach was used to design a new architecture for brain tumor classification that was tested using two openly accessible datasets from Figshare and Kaggle [31]. The authors presented a method for brain tumor detection that uses a low-complexity architecture to reduce the processing time of a traditional RCNN structure. Firstly, to identify glioma and healthy tumor MRI images, they used a Two-Channel CNN, a low-complex framework, which improves accuracy by 98.21%. Afterward, this framework is employed as a feature extractor in an RCNN to identify tumor areas in a Glioma MRI dataset that is categorized from a preceding phase. Lastly, the tumor region is bounded by boxes. This approach has been used for two more tumor types: meningioma and pituitary tumors. With an overall confidence level of 98.8%, their approach could achieve a low execution time in comparison to state-of-the-art techniques.

ImageNet-based Vision Transformer (ViT) models (B/16, B/32, L/16, and L/32) that have been trained and fine-tuned were proposed by [32] for brain tumor classification purposes. Validation and testing were performed on a three-classes brain tumor dataset from figshare that included 3064 T1w contrast-enhanced (CE) MRI slices with gliomas, meningiomas, and pituitary tumors. L/32 was the highest model, gaining 98.2% in the total test accuracy at a resolution of 384×384. The ensembles of all four ViT algorithms showed an average testing accuracy of 98.7% at the same resolution, surpassing the performance of each algorithm at both resolutions and their ensembling at resolution 224×224.

### 2.2. Machine Learning-Based Techniques

Pareek et al. [33] presented a method that detects if there is a tumor or not and then classifies the tumor type. The proposed method was tested on 150 T1-weighted MRI brain imaging for identifying brain tumors. The supervised approach was utilized for the classification process, and the principal component analysis was employed for feature extraction. They also assessed the tumor’s area and volume to determine the tumor’s levels. The findings of the experiments demonstrate that KSVM is 97 percent accurate in classifying brain tumors.

A novel method proposed in [34] produces excellent results and outperforms earlier techniques. To improve MRI quality and to build an exclusionary feature set, the suggested method employs normalization, densely speeded-up powerful features, and histogram of gradient approaches. In the classifying stage, they use a support vector machine. The proposed system has been tested on a significant dataset. The accuracy obtained with this method is 90.27 percent compared to state-of-the-art techniques. Regarding experimental findings, this strategy outperformed the most recent techniques. These findings were obtained by a rigorous statistical study (k-fold cross-validation), demonstrating the recommended method’s accuracy and robustness.

A quantum Fully Self neural network (QFS-Net) network using qubits/three states of quantum for segmentation of the brain lesions has been proposed [35] as a method to benefit from the capabilities of quantum correlations. The advanced quantum back-propagation approach used in supervised QINN networks is replaced with a ground-breaking supervised qutrit-based counter-propagating technique in the QFS-Net. This method enables the propagation of iterative quantum states throughout the network’s layers.

### 2.3. Hybrid-Based Techniques

Khairandish et al. [36] Presented a hybrid approach that combines CNN and SVM with threshold-based segmentation in terms of classification. The hybrid proposed CNN-SVM demonstrates enhanced overall accuracy with 98.4959%. To extract features from tumor regions and adjacent tissues, pre-trained AlexNet, GoogLeNet, ShuffleNet, and ResNet-18 networks are employed [37]. Although deep features are crucial in the identification process, some low-level data about tumors may be lost. As a result, a shallow network is made to learn low-level data. Deep and shallow features are blended to compensate for the loss of data. With the fused feature sets, SVM and k-NN classifiers are trained. Data augmentation and ROI expansion simultaneously enhance the average sensitivity by roughly 11.72 percent, according to experimental results. These findings support the theory that the tissues around the tumor contain significant data. Not only that, but feature fusion may substitute for missing low-level information. Furthermore, the deep feature extractor process is conducted with the ResNet-18. their experimental results are competitive compared to state-of-the-art techniques.

Authors in [38] proposed a deep learning-based automatic multimodal classification technique for categorizing different brain tumors. The suggested approach comprises five essential phases. In the first phase, an edge-based histogram and the Discrete Cosine Transform (DCT) are used to implement the linear contrasting stretching. Phase two involves the deep learning component Extractions are made. Deep learning feature extraction is performed in the second phase. Two pre-CNN networks, namely VGG16 and VGG19, were employed for feature extraction. The Extreme Learning Machine (ELM) and a correntropy-based strategy were both employed in the third stage to choose the best features. The resistant covariant features based on Partial Least Squares (PLS) were combined into one matrix. ELM received the merged matrix to conduct the classification model. The suggested technique was tested using the three datasets (BraTs2015, BraTs2017, and BraTs2018) with an accuracy of 97.8%, 96.9%, and 92.5%, respectively.

A hybrid deep learning-based technique to classify brain tumors with ISLES2015 and BRATS2015 datasets was proposed in [39]. DLS techniques such as VGG16, VGG19, and ResNet50 are used for experimental results. Then, the classifiers SoftMax, SVM-RBF, and SVM-Cubic are used to construct the multi-class classification, and performance is calculated according to the total accuracy reached by each method. The results of this study proved that VGG19 with SVM-Cubic has significantly greater accuracy (96%) than other methods.

Irmak et al. [40] proposed three-distinct Convolutional networks are suggested for three distinct classification architectures. Detection of brain tumor accuracy reaches 99.33 percent in the first CNN architecture. The accuracy of the second CNN model architecture reaches approximately 92.66 percent. The second CNN architecture can categorize brain tumors into five types: normal, meningioma, glioma, metastatic, and pituitary. With an accuracy of 98.14 percent, the third CNN architecture successfully categorizes brain tumors into Grade II, Grade III, and Grade IV. The state-of-the-art CNN algorithms such as Inceptionv3, AlexNet, ResNet-50, GoogleNet, and VGG-16 compared to the suggested CNN models. Utilizing the grid search optimization technique, all the essential model parameters of Convolutional networks are automatically identified. Publicly released clinical datasets are used to generate acceptable detection results.

**Table 1:** Different brain tumor classification techniques.

Reference	Used Technique	Dataset	Accuracy	Advantages	Disadvantages
[26]	16-layer VGG-16 deep NN	Hospitals’ dataset from 2010–2015, China	98%	Improve multi-class brain tumor classification accuracy.	Small dataset
[27]	CNN-based DL model	REMBRANDT	100% for two-class classification	AI-based transfer learning surpasses machine learning for brain tumor classification.	—
[28]	CNN technique for a three-class classification	Figshare	98%	Transfer learning techniques is a highly effective strategy when medical pictures are scarce.	Samples from the category meningioma were misclassified. overfitting with smaller training data.
[31]	RCNN-based model	Openly accessible datasets from Figshare and Kaggle	98.21%	Low execution time that is optimal for real time processing. Operate on limited-resources systems	Limited to object detection and need to implement brain segmentation.
[36]	Hybrid CNN-SVM	BRATS 2015	98.49%	Provide effective classification technique for brain tumor	Need to consider the size and location of brain tumor.
[37]	SVM and k-NN classifiers	Figshare, 2017	97.25%	Extended ROI’s shallow and deep properties improve classifier performance	Accuracy need to be improved
[29]	Deep inception residual network	Publicly accessible brain tumor imaging dataset with 3064 pictures	99.69%	Achieves high classification performance.	Large number of parameters. Maximum computational time.
[40]	CNN model for multi-classification	Publicly released clinical datasets	99.33%	CNN models can help doctors and radiologists validate their first brain tumor assessment	—
[33]	Kernel-based SVM	Figshare	97%	Can detect whether brain tumor is benign and malignant.	Small dataset. Classification accuracy need to be increased.
[30]	Transfer learning-based classification	Figshare	99.02%	Accurately detect brain tumors. Transfer learning in healthcare can help doctors make quick, accurate decisions.	Classification accuracy need to be increased.
[34]	Multi-classification model	Three different publicly datasets	90.27%	Low computational time. Help doctors in making better classification decisions for brain cancers.	Classification accuracy need to be increased.
[32]	ImageNet-based ViT	Figshare	98.7%	Accurately detect brain tumors. Helps radiologists make the right patient-based decision	Needs to consider the size and location of brain tumor
[38]	Deep learning-based automatic multimodal classification	BraTs 2015, BraTs 2017, BraTs 2018	97.8%	Feature extraction improved classification accuracy and reduced processing time	Classification accuracy need to be increased
[39]	Hybrid deep learning-based	ISLES2015 and BRATS2015	96%	Perform multi-class classification for brain tumor	Classification accuracy need to be increased

## 3. Brain Tumor Classification Model Based CNN (BCM-CNN)

**Figure 1:**
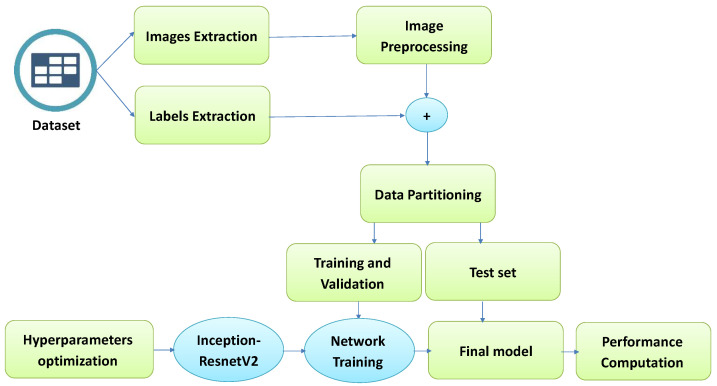
The proposed BCM-CNN model steps.

This section proposes a Brain Tumor Classification Model (BCM-CNN) based on an advanced model using a Convolutional Neural Network. The overall architecture of the proposed model is shown in Figure 1. The BCM-CNN is used to diagnose a brain tumor. It consists of a hyperparameters optimization, followed by an Inception-ResnetV2 training model. The model’s output is a binary 0 or 1 (0: Normal, 1: Tumor) and uses common pre-trained models (Inception-ResnetV2) to enhance the brain tumor diagnosis process.

### 3.1. CNN Hyperparameters Optimization

This subsection discusses the selected hyperparameters. The configuration’s hyperparameters are variables that are not part of the model and whose values cannot be inferred from the data. Two main categories of hyperparameters exist: (i) a network structure-determining hyperparameter; (ii) the network is trained by the hyperparameter. Table 2 contains a list of the hyperparameters that were examined in this study.

### 3.2. ADSCFGWO for CNN Hyperparameters

To select the most important characteristics from the metamaterial dataset in order to achieve the best possible performance, the adaptive dynamic sine cosine fitness grey wolf optimizer, abbreviated as ADSCFGWO, was initially introduced in [41]. This algorithm draws from both the sine cosine and grey wolf algorithms in an adaptable framework that makes use of both algorithms’ strengths. To estimate the double T-shape monopole antenna properties, the ADSCFGWO algorithm additionally optimizes a bidirectional recurrent neural network (BRNN). In this work, the optimization of the CNN hyperparameters is based on the adaptive dynamic sine cosine fitness grey wolf optimizer (ADSCFGWO) algorithm. The ADSCFGWO algorithm is shown in Algorithm 1.

The population in the potential solution of the ADSCFGWO algorithm, $X_{i}\left(i=1,2,\ldots ,n\right)$ with size *n*, is split into two groups: the exploration group, $n_{1}$, and exploitation group, $n_{2}$. The exploration group’s job is to use the search space to discover new locations where the greatest possible solution might be located. The exploitation group’s job is to use an objective function to enhance the best solution’s quality. These two groups cooperate in the suggested optimization process to trade responsibilities and required data that can hasten the retrieval of the optimum solution. The effective avoidance of the local optima and the precise exploration of the search space are advantages of this collaboration. The ADSCFGWO optimization technique has two key characteristics: first, it maintains correct control over the equilibrium between the exploitation and exploration groups; and second, it uses a dynamic mechanism to avoid steady regions in the search space.

The fittest solutions are denoted by $\left(S_{\alpha }\right)$, $\left(S_{\beta }\right)$, and $\left(S_{\delta }\right)$. The position update in the direction of the prey position is estimated during the search process as

(1) $$X\left(t+1\right)=\frac{T_{1}+T_{2}+T_{3}}{3}$$

where $T_{1},T_{2},and\;T_{3}$ are calculated as

(2) $$T_{1}=S_{\alpha }-A_{1}.D,\;T_{2}=S_{\beta }-A_{2}.D,\;T_{3}=S_{\delta }-A_{3}.D$$

where *D* is calculated as $|C_{1}.\left(F_{\alpha }*S_{\alpha }+F_{\beta }*S_{\beta }+F_{\delta }*S_{\delta }\right)-X\left(t\right)|$. The *A* and *C* vectors are defined as $A=2a.r_{1}-a$ and $C=2r_{2}$, where the vectors values $r_{1}$ and $r_{2}$ are randomly selected from the range [0,1]. The values of *a* are determined in the range [0,2] and is calculated as $a=2-t.\frac{2}{T_{Max}}$ for $T_{Max}$ iterations. The fitness functions are calculated as

(3) $$F_{\alpha }=\frac{F_{\alpha }}{F_{\alpha }+F_{\beta }+F_{\delta }},\;F_{\beta }=\frac{F_{\beta }}{F_{\alpha }+F_{\beta }+F_{\delta }},\;F_{\delta }=\frac{F_{\delta }}{F_{\alpha }+F_{\beta }+F_{\delta }}$$

The ADSCFGWO method automatically balances the subgroups of the population’s exploitation and exploration. The algorithm uses a 70/30 system in which two groups—exploration and exploitation groups—represent 70% of the population. A large number of participants in the exploration group early in the optimization process helps with the discovery of novel and intriguing search regions. The overall fitness of agents increases when more exploitative agents can increase their fitness values, but the proportion of agents engaged in exploration falls quickly from 70% to 30%. If a better solution cannot be identified, using an elitism approach ensures convergence by keeping the process leader in consecutive populations. ADSCFGWO may at any point increase the size of the exploration group, provided that the leader’s fitness has not dramatically increased over the course of three consecutive iterations.

The suggested ADSCFGWO algorithm’s computational complexity can be stated as in Table 3 for population *n* and iterations $t_{max}$. From this analysis, the complexity of computations is $O(t_{max}\times n)$ and $O(t_{max}\times n\times d)$ with *d* dimension.
**Algorithm 1** ADSCFGWO algorithm1:**Initialize** ADSCFGWO population $X_{i}\left(i=1,2,\ldots ,n\right)$ with size *n*, iterations $t_{Max}$, fitness function $F_{n}$, parameters (*a*, $A_{1}$, $A_{2}$, $A_{3}$, $C_{1}$, $C_{2}$, $r_{1}$, $r_{2}$, $r_{3}$, $r_{4}$)2:**Calculate** fitness function $F_{n}$ for each $X_{i}$3:**Find** best solutions as $S_{\alpha },S_{\beta },S_{\delta }$4:**Set** t = 15:**while**$\;t\leq t_{Max}$**do**6:  **Update** $r_{1}$ by $r_{1}=a\left(1-\frac{t}{Max_{iter}}\right)$7:  **for** ($i=1:i\leq n_{1}$) **do**8:      **DynamicSearch**($F_{n}$)9:      **Update** Fitness by Equation (3)10:    **Update** positions from GWO as $X\left(t+1\right)=\frac{T_{1}+T_{2}+T_{3}}{3}$11:    **if** ($r_{4}<0.5$) **then**12:      **Update** positions from SCA as $X\left(t+1\right)=X\left(t\right)+r_{1}\times \sin\left(r_{2}\right)\times \left|r_{3}S_{\alpha }-X\left(t\right)\right|$13:    **end if**14:  **end for**15:  **for** ($i=1:i\leq n_{2}$) **do**16:    **DynamicSearch**($F_{n}$)17:    **Update** Fitness by Equation (3)18:    **Update** positions from GWO as $X\left(t+1\right)=\frac{T_{1}+T_{2}+T_{3}}{3}$19:    **if** ($r_{4}\geq 0.5$) **then**20:      **Update** positions from SCA as $X\left(t+1\right)=X\left(t\right)+r_{1}\times \cos\left(r_{2}\right)\times \left|r_{3}S_{\alpha }-X\left(t\right)\right|$21:    **end if**22:  **end for**23:  **Update** fitness function $F_{n}$ for each $X_{i}$24:  **Update** parameters25:  **Find** best solutions as $S_{\alpha },S_{\beta },S_{\delta }$26:**end while**27:**Return** best solution $X^{*}$28:**DynamicSearch**($F_{n}$)29:**if** (Best $F_{n}$ is same for three iterations) **then**30:  **Increase** exploration group solutions $\left(n_{1}\right)$31:  **Decrease** exploitation group solutions $\left(n_{2}\right)$33:**end if**

### 3.3. 3D U-Net Architecture Segmentation Model

U-Net [42] is a network that is used for fast and accurate image segmentation. It comprises an expanded pathway and a contracting pathway. The contracting pathway adheres to the standard convolutional network design. Two 3×3 unpadded convolution layers are applied repeatedly, and after them, a ReLU activation function and a 2×2 max-pooling with stride 2 are applied for down-sampling. The number of features at every stage in the down-sampling process is doubled. The expanding pathway consists of an up-sampling process, a 2×2 convolution layer that reduces the size of the feature map, a combination with the proportionally clipped feature map from the contracting pathway, and two 3×3 convolution layers, each accompanied by a ReLU activation function. All the 64-component extracted features are mapped to the required number of categories in the last layer using a 1×1 convolution layer. The model includes 23 convolutional layers overall. The reason for using a U-net network is that it is fast compared to other networks. On a modern GPU, segmentation of a 512×512 picture consumes less than a second.

**Figure 2:**
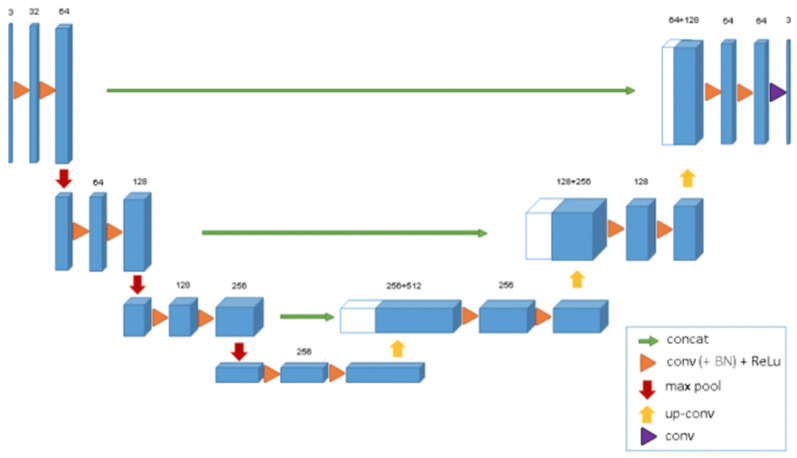
Structure of the 3D U-Net.

Numerous U-Net-based variant networks have been proposed since U-Net [42]’s extensive research and application in medical image segmentation in 2015; 3D U-Net [43] is the most representative of these. Figure 2 depicts the 3D U-Net’s structure. The encoder-decoder architecture of this model expands on the prior U-Net (2D). The encoder component performs feature extraction from an analysis of the input image. The associated decoder produces a segmented mask. The mask extraction is supervised by this model by minimizing a cost function. 3D U-Net differs from 2D U-Net in that its features are extracted and restored using 3D convolution, 3D max-pooling, and 3D deconvolution blocks in turn after the volume data are input. In addition, batch normalization is added by 3D U-Net to prevent bottlenecks and hasten convergence. For the segmentation process, the dataset is partitioned into a train, validation, and test datasets.

## 4. Experimental Results

This section describes the used dataset, the Performance metrics used in CNN, the implementation of the proposed strategy, and the experiments conducted. The parameters for the ADSCFGWO algorithm’s configuration are shown in Table 4.

### 4.1. Dataset Description

**Figure 3:**
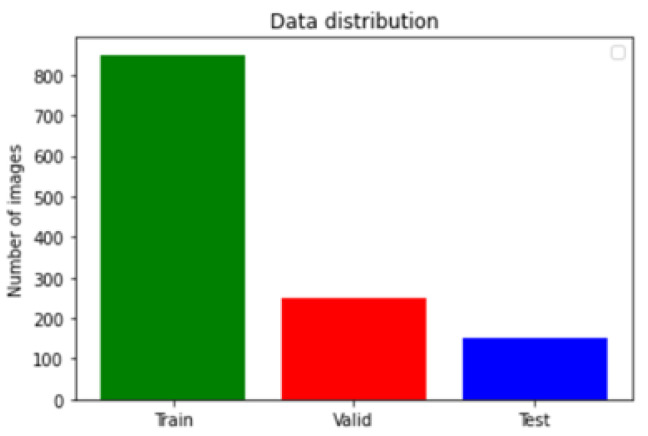
Dataset distribution.

**Figure 4:**
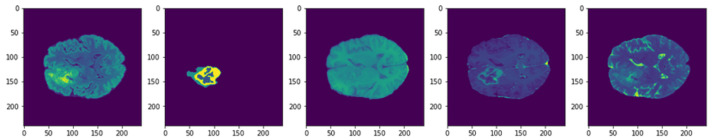
Example of dataset images.

The used dataset is BRaTS 2021 Task 1 Dataset [44]. As training, validation, and testing data for this year’s BraTS challenge, a sizable number of multi-institutional regular clinically acquired multi-parametric MRI (mpMRI) images of glioma with pathologically confirmed diagnosis and accessible MGMT promoter methylation status are used. For Task 1, the datasets utilized in this year’s competition have been updated with many additional routines clinically collected mpMRI scans since BraTS’20. To quantitatively assess the projected tumor segmentations, expert neuroradiologists create and approve ground truth annotations of tumor sub-regions for each patient in the training, validation, and testing datasets. As shown in Figure 3, the dataset is partitioned into a train, validation, and test datasets. Figure 4 illustrates an example of the dataset.

The data augmentation technique is used in this study to artificially generate fresh training data from the current data. As a sort of data augmentation, picture augmentation produces altered representations of the training dataset’s images. The input dataset is subjected to several image transformations, such as horizontal and vertical shift, horizontal and vertical flip, random rotation, and random zoom. The shift augmentation maintains the same image dimensions while shifting all of the MRI image’s pixels in either a horizontal or vertical direction. When flipping horizontally or vertically, all pixels’ rows and columns are reversed. The MRI image is randomly rotated between 0 and 360 degrees clockwise using the rotation augmentation. The zoom augmentation’s final step involves randomly zooming the MRI image by a factor between [0.9, 1.1].

### 4.2. Performance Metrics Used in CNN

The conventional computer-aided diagnostic approach may be tested using a variety of key performance metrics, including accuracy, precision, F1-score, recall, specificity, and sensitivity. The number of cases that were accurately identified as defective is shown by the letter TP, which stands for True Positive. False Positive, abbreviated as FP, refers to the number of cases that were incorrectly identified as defective. Additionally, FN stands for False Negative and reflects the number of occurrences that were incorrectly classified as non-defective. TN is for True Negative, which represents the number of cases that were correctly identified as non-defective. The metrics are defined as in Table 5.

### 4.3. The BCM-CNN Evaluation

As shown in Table 6, the effectiveness of the suggested approach (BCM-CNN) is evaluated in comparison to the previously widely used classifiers CNN [22], Decision Tree (DT) [45], Linear Discriminant (LD) [46], Support Vector Machine (SVM) [47], and K-Nearest Neighbor (K-NN) [48]. The default parameters are used for these methods.

Samples from the texted dataset are employed in the classification experiment. As a result of the BCM-CNN based on the ADSCFGWO algorithm boosting the performance of the CNN after altering its hyperparameters, the BCM-CNN delivered the best results when employed as a classifier, with an accuracy of (0.99980004), Sensitivity (TRP) of (0.99980004), Specificity (TNP) of ( 0.99980004), Pvalue (PPV) of (0.99980004), Nvalue (NPV) of (0.99980004), and F1-score of (0.9998). After the SVM-Linear model, which has an accuracy score of (0.968992248), the K-NN model, which has an accuracy score of (0.965250965), and finally, the LD model, which has an accuracy score of (0.961538462), the simple CNN model gets the second-best accuracy with a score of (0.9765625). The DT model was only able to achieve the lowest level of accuracy with (0.956022945). This came about as a consequence of the fact that the method that was proposed resulted in an improvement in CNN’s overall performance.

Table 7 shows the proposed BCM-CNN-based classifier’s statistical description and a comparison of classifiers based on 11 runs (run the algorithm 11 times) and 80 iterations ($t_{Max}$ in Algorithm 1) for 10 agents (Population size *n* in Algorithm 1) of the ADSCFGWO algorithm. This is to confirm the stability of the proposed method compared to other methods. Table 8 presents the compared and the proposed classifier’s test results using a one-way ANOVA (analysis of variance) test. In contrast, Table 9 discusses the comparison and the proposed classifiers test results using the Wilcoxon Signed-Rank test. With a p-value of less than 0.05, this statistical test demonstrates the significant difference between the suggested BCM-CNN classifier’s results and those of other classifiers.

**Figure 5:**
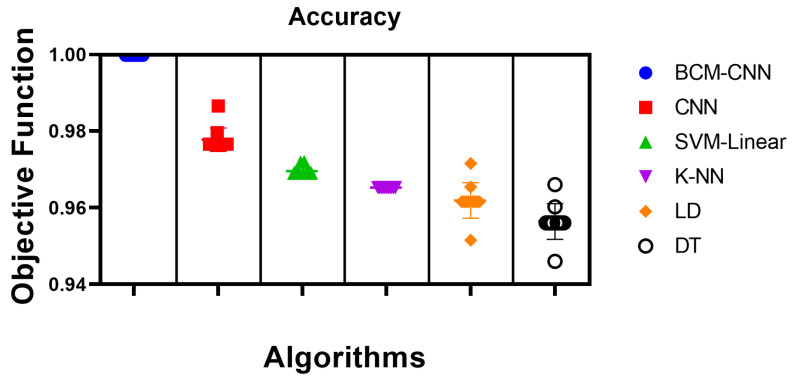
Box plot of accuracy for the BCM-CNN model under consideration and the contrasting models.

**Figure 6:**
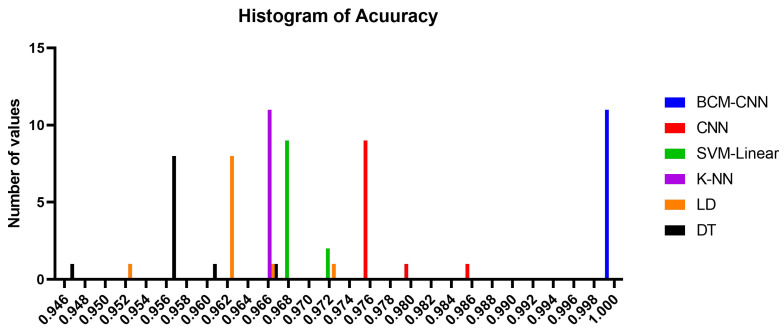
Histogram of accuracy for the BCM-CNN model under consideration and the contrasting models.

The accuracy of the proposed BCM-CNN and comparative methods is shown by the box plot in Figure 5. This graph demonstrates the maximum accuracy results that the BCM-CNN-based optimization algorithm was able to produce. Based on the number of values with the Bin Center range (0.946–1.0), the accuracy histogram for the algorithms that have been presented and compared is shown in Figure 6, which attests to the stability of the suggested algorithm.

**Figure 7:**
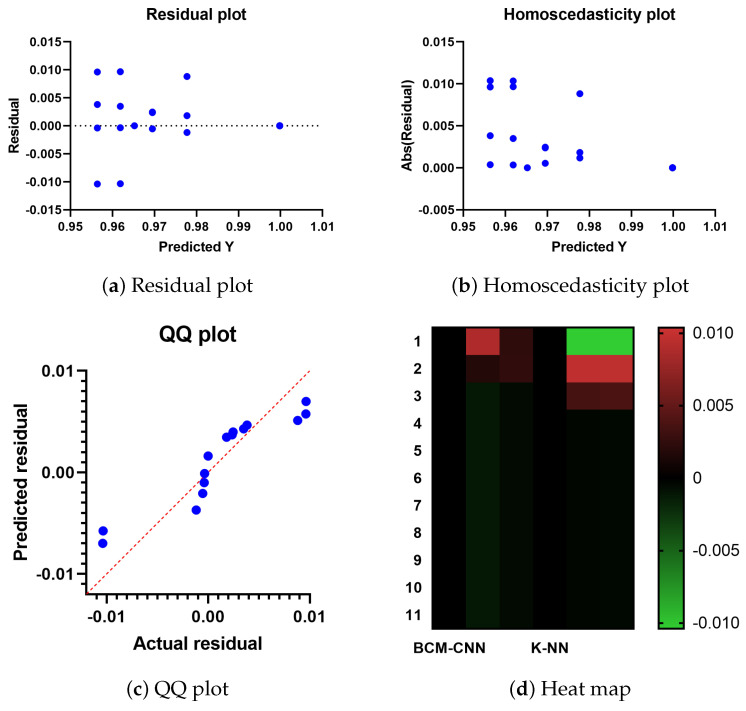
The heat map, residual, QQ, and homoscedasticity plots of the ADSCFGWO and comparable algorithms.

Figure 7 displays the residual, QQ (quantile-quantile), homoscedasticity plots, and heat map for the proposed and compared techniques. The possible problems can be observed in the residual values and plots as opposed to the plot of the original dataset. The independent variable is plotted on the horizontal axis, while the residual values are plotted on the vertical axis. The ideal situation is achieved if the residual values are scattered randomly and uniformly along the horizontal axis. The residual value is calculated as follows when the mean and the sum of the residuals are both equal to zero: (Actual-Predicted values). Figure 7 displays the residual plot. To determine if a model is linear or nonlinear and which one is best, plot patterns in a residual plot can be used. The projected scores for the dependent variable are examined visually together with the homogeneity of variance or heteroscedasticity. When the error term, also known as noise or random disturbance in the relationship between the dependent and independent variables, is constant across all values of the independent variables, this situation is referred to as homoscedasticity. The heteroscedasticity plot, shown in Figure 7, improves the precision of the research results. Any infraction can be quickly and easily detected.

The QQ plot is also shown in Figure 7. A probability plot is one illustration. By plotting the quantiles against one another, two probability distributions are primarily compared. It is possible to see that the point distributions in the QQ plot fit on the line in the illustration. Since the relationship between the actual and projected residuals is linear, the suggested technique is effective. Figure 7 serves as a tool for data visualization and displays heat maps for the offered and contrasted algorithms. The intensity of a two-dimensional color scale indicates the complexity of an algorithm. The color fluctuation provides obvious visual cues as to how the proposed solution is superior to the comparable algorithms. The ADSCFGWO algorithm’s performance in feature selection, as seen in Figure 7, is supported by these figures.

### 4.4. 3D U-Net Segmentation Model

**Figure 8:**
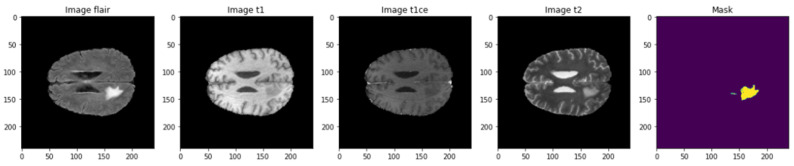
Visualize image with mask of a positive brain tumor.

**Figure 9:**
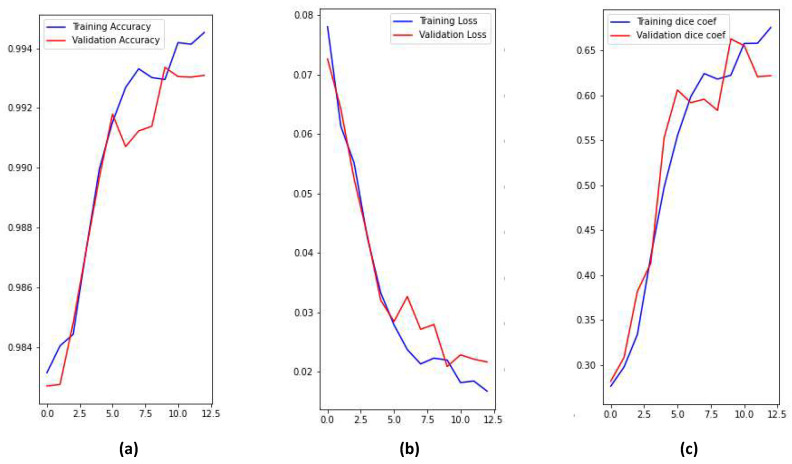
Training and validation performance parameters; (**a**) Accuracy, (**b**) Loss, (**c**) Dice coef.

There are four classes in the segmentation process. Segmentation classes are NOT tumor, non-enhancing tumor (RED color), EDEMA (Green color), and ENHANCING (yellow color). These classes were converted into three classes later. Figure 8 illustrates samples of images and masks with a positive brain tumor. For more precious and fast detection of brain tumor, the 3D U-net segmentation model has been implemented on the BRaTS 2021 dataset. The dataset is divided into 70% training, 20% validation, and 10% testing. Implementation is constructed online on Kaggle. U-net model enhances segmentation validation accuracy up to 99.33%, and validation loss up to 0.01 as shown in Figure 9. We can conclude that our proposed model can detect brain tumor with high accuracy compared to state-of-the-art techniques in terms of classification and segmentation.

## 5. Conclusions and Future Work

A large number of researchers looked at a wide variety of algorithms with the goal of accurately detecting and classifying brain cancers in a quick and efficient manner. Deep learning (DL) makes it possible to use a Convolutional Neural Network (CNN) model that has already been pre-trained for the analysis of medical pictures, in particular for the categorization of brain tumors. The fundamental objective of this research is to develop an improved model with the intention of making brain tumor diagnosis more accurate. A Convolutional Neural Network (CNN) that is based on a Brain Tumor Classification Model (BCM-CNN) was proposed in this paper. The optimization of the CNN’s hyperparameters was based on an adaptive dynamic sine-cosine fitness grey wolf optimizer (ADSCFGWO) algorithm. The BCM-CNN was used as a classifier in the experiments, and the results reveal that it produced the best results due to the enhancement of the performance of the CNN after the optimization was performed. The BCM-CNN was given the BRaTS 2021 Task 1 dataset, and it performed with an accuracy of 99.99%. The main limitation of the proposed algorithm is that it takes a long time to process due to the extra optimization steps. It may not be applicable as the size of the trained data is limited, so we intend to solve this issue in future work by generalizing more data. We also intend to make a prediction in future work, not just classification.

## Figures and Tables

**Table 2:** CNN hyperparameters setup.

Parameter	Value
**CNN training options (Default)** RateDropFactor Momentum Learn L2Regularization LearnRateDropPeriod GradientThreshold GradientThresholdMethod ValidationData VerboseFrequency ValidationPatience ValidationFrequency ResetInputNormalization **CNN training options (Custom)** InitialLearnRate ExecutionEnvironment MiniBatchSize MaxEpochs Verbose Shuffle LearnRateSchedule Optimizer	0.1000 0.9000 1.0000 $\times 10^{-4}$ 10 Inf l2norm imds 50 Inf 50 1 1.0000 $\times 10^{-4}$ gpu 8 20 0 every-epoch piecwise ADSCFGWO

**Table 3:** ADSCFGWO algorithm’s computational complexity.

No.	Operation	Complexity
1	Initialization	O(1)
2	Calculate objective function	O(n)
3	Finding best solutions	O(n)
4	Updating position of current grey wolf by fitness	$O(t_{max}\times n)$
5	Updating position of current individual by Sine Cosine	$O(t_{max}\times n)$
6	Updating objective function	$O(t_{max}\times n)$
7	Finding best fitness	$O(t_{max})$
8	Updating parameters	$O(t_{max})$
9	Producing the best fitness	O(1)

**Table 4:** Parameters for the ADSCFGWO algorithm’s configuration.

Parameter	Value
a	[0, 2]
$r_{1}$,$r_{2}$,$r_{3}$,$r_{4}$	[0, 1]
# Runs (Repeat the whole algorithm)	11
# Iterations ($t_{Max}$)	80
# Agents (Population size *n*)	10

**Table 5:** Performance metrics used in CNN.

No.	Metrics	Calculation
1	Accuracy	$\frac{TP+TN}{TP+TN+FP+FN}$
2	Sensitivity	$\frac{TP}{TP+FN}$
3	Specificity	$\frac{TN}{TN+FP}$
4	Precision (PPV)	$\frac{TP}{TP+FP}$
5	Negative Predictive Value (NPV)	$\frac{TN}{TN+FN}$
6	F1-score	$2\times \frac{PPV\times TPR}{PPV+TPR}$

**Table 6:** The performance of the proposed method (BCM-CNN) versus basic classifiers.

	Accuracy	Sensitivity (TRP)	Specificity (TNP)	*p* Value (PPV)	N Value (NPV)	F1-Score
BCM-CNN	0.99980004	0.99980004	0.99980004	0.99980004	0.99980004	0.9998
CNN	0.9765625	0.975609756	0.977198697	0.966183575	0.983606557	0.970874
SVM-Linear	0.968992248	0.956937799	0.977198697	0.966183575	0.970873786	0.961538
K-NN	0.965250965	0.956937799	0.970873786	0.956937799	0.970873786	0.956938
LD	0.961538462	0.947867299	0.970873786	0.956937799	0.964630225	0.952381
DT	0.956022945	0.947867299	0.961538462	0.943396226	0.964630225	0.945626

**Table 7:** Proposed BCM-CNN classifier’s statistical description and a comparison of classifiers.

	BCM-CNN	CNN	SVM-Linear	K-NN	LD	DT
Number of values	11	11	11	11	11	11
Minimum	0.9998	0.9766	0.969	0.9653	0.9515	0.946
25% Percentile	0.9998	0.9766	0.969	0.9653	0.9615	0.956
Median	0.9998	0.9766	0.969	0.9653	0.9615	0.956
75% Percentile	0.9998	0.9766	0.969	0.9653	0.9615	0.956
Maximum	0.9998	0.9866	0.972	0.9653	0.9715	0.966
Range	0	0.01	0.003	0	0.02	0.02
10% Percentile	0.9998	0.9766	0.969	0.9653	0.9535	0.948
90% Percentile	0.9998	0.9852	0.972	0.9653	0.9703	0.9649
95% CI of median						
Actual confidence level	98.83%	98.83%	98.83%	98.83%	98.83%	98.83%
Lower confidence limit	0.9998	0.9766	0.969	0.9653	0.9615	0.956
Upper confidence limit	0.9998	0.9796	0.9719	0.9653	0.9654	0.9602
Mean	0.9998	0.9777	0.9695	0.9653	0.9619	0.9564
Std. Deviation	0	0.00306	0.001195	0	0.00462	0.004649
Std. Error of Mean	0	0.0009226	0.0003603	0	0.001393	0.001402
Lower 95% CI of mean	0.9998	0.9757	0.9687	0.9653	0.9588	0.9533
Upper 95% CI of mean	0.9998	0.9798	0.9703	0.9653	0.965	0.9595
Coefficient of variation	0.000%	0.3130%	0.1232%	0.000%	0.4803%	0.4860%
Geometric mean	0.9998	0.9777	0.9695	0.9653	0.9619	0.9564
Geometric SD factor	1	1.003	1.001	1	1.005	1.005
Lower 95% CI of geo. mean	0.9998	0.9757	0.9687	0.9653	0.9588	0.9533
Upper 95% CI of geo. mean	0.9998	0.9798	0.9703	0.9653	0.965	0.9595
Harmonic mean	0.9998	0.9777	0.9695	0.9653	0.9619	0.9564
Lower 95% CI of harm. mean	0.9998	0.9757	0.9687	0.9653	0.9588	0.9533
Upper 95% CI of harm. mean	0.9998	0.9798	0.9703	0.9653	0.965	0.9595
Quadratic mean	0.9998	0.9777	0.9695	0.9653	0.9619	0.9564
Lower 95% CI of quad. mean	0.9998	0.9757	0.9687	0.9653	0.9588	0.9533
Upper 95% CI of quad. mean	0.9998	0.9798	0.9703	0.9653	0.965	0.9595
Skewness		2.887	1.924		−0.2076	−0.2118
Kurtosis		8.536	2.047		4.001	3.839
Sum	11	10.76	10.66	10.62	10.58	10.52

**Table 8:** Compared and proposed classifiers test results for ANOVA.

	SS	DF	MS	F (DFn, DFd)	*p* Value
Treatment (between columns)	0.01323	5	0.002646	F (5, 60) = 295.4	*p* < 0.0001
Residual (within columns)	0.0005374	60	0.000008957	-	-
Total	0.01377	65	-	-	-

**Table 9:** Compared and proposed classifiers test results for Wilcoxon Signed-Rank.

	BCM-CNN	CNN	SVM-Linear	K-NN	LD	DT
Theoretical median	0	0	0	0	0	0
Actual median	0.9998	0.9766	0.969	0.9653	0.9615	0.956
Number of values	11	11	11	11	11	11
Wilcoxon Signed Rank Test						
Sum of signed ranks (W)	66	66	66	66	66	66
Sum of positive ranks	66	66	66	66	66	66
Sum of negative ranks	0	0	0	0	0	0
P value (two tailed)	0.001	0.001	0.001	0.001	0.001	0.001
Exact or estimate?	Exact	Exact	Exact	Exact	Exact	Exact
Significant (alpha = 0.05)?	Yes	Yes	Yes	Yes	Yes	Yes
How big is the discrepancy?						
Discrepancy	0.9998	0.9766	0.969	0.9653	0.9615	0.956

## Data Availability

Not applicable.

## Abbreviations

The following abbreviations are used in this manuscript:

AI	Artificial intelligence
DL	Deep Learning
CNN	Convolutional Neural Network
BCM-CNN	Brain Tumor Classification Model based on CNN
ADSCFGWO	Adaptive Dynamic Sine-Cosine Fitness Grey Wolf Optimizer
WHO	World Health Organization
MEG	Magneto Encephalon Graph
CT	Computed Tomography
EEG	Ultrasonography, Electro Encephalon Graph
SPECT	Single-Photon Emission Computed Tomography
PET	Positron Emission Tomography
MRI	Magnetic Resonance Imaging
CAD	Computer-Aided Diagnosis
NN	Neural Network
FC	Fully Connected
DT	Decision Tree
NB	Naive Bayes
LD	Linear Discrimination
SVM	Support Vector Machine
AUC	Area Under the Curve
LD	Linear Discriminant
QQ	Quantile-Quantile
